# Mechanical and Metallurgical In Vitro Evaluation of Electropolished Versus Non‐Electropolished Rotary and Reciprocating Instruments

**DOI:** 10.1111/iej.70009

**Published:** 2025-08-04

**Authors:** Victor T. L. Vieira, Emmanuel J. N. L. Silva, Jorge N. R. Martins, Alejandro Jaime, Carlos Garcia Puentes, António Roma Torres, Francisco Manuel Braz Fernandes, Marco A. Versiani, Gustavo De‐Deus, Felipe G. Belladonna

**Affiliations:** ^1^ School of Dentistry Grande Rio University (UNIGRANRIO) Rio de Janeiro Brazil; ^2^ Deparment of Endodontics Fluminense Federal University Niterói Brazil; ^3^ Department of Endodontics Rio de Janeiro State University Rio de Janeiro Brazil; ^4^ Faculdade de Medicina Dentária Universidade de Lisboa Lisboa Portugal; ^5^ Grupo de Investigação em Bioquimica e Biologia Oral, Unidade de Investigação em Ciências Orais e Biomédicas (UICOB), Faculdade de Medicina Dentária Universidade de Lisboa Lisboa Portugal; ^6^ Centro de Estudo de Medicina Dentária Baseada na Evidência (CEMDBE) – Cochrane Portugal, Faculdade de Medicina Dentária Universidade de Lisboa Lisboa Portugal; ^7^ LIBPhys‐FCT UID/FIS/04559/2013, Faculdade de Medicina Dentária Universidade de Lisboa Lisboa Portugal; ^8^ Department of Endodontics Maimonides University (UMAI) Buenos Aires Argentina; ^9^ Private Practice Porto Portugal; ^10^ CENIMAT/I3N, Department of Materials Science NOVA School of Science and Technology Universidade NOVA de Lisboa Caparica Portugal; ^11^ Dental Specialty Center, Brazilian Military Police Belo Horizonte Minas Gerais Brazil

**Keywords:** electropolishing, endodontics, mechanical properties, nickel‐titanium instruments, reciprocating instrument, rotary instrument

## Abstract

**Aim:**

To evaluate the effect of electropolishing on the mechanical properties of One RECI and One Curve mini nickel‐titanium (NiTi) instruments by comparing electropolished and non‐electropolished versions of each instrument type.

**Methodology:**

Electropolished and non‐electropolished One RECI (reciprocating) and One Curve mini (rotary) NiTi instruments, all manufactured with identical geometry and heat treatment, were evaluated. Instrument design was analysed by light microscopy and scanning electron microscopy, while metallurgical characterisation was performed using energy‐dispersive X‐ray spectroscopy (EDS) and differential scanning calorimetry (DSC). Mechanical performance was assessed through torsional resistance, bending and buckling load, surface microhardness, and cutting efficiency. Statistical comparisons were performed using the independent samples t‐test or the Mann–Whitney *U*‐test, with significance set at *p* < 0.05.

**Results:**

Design and metallurgical analyses confirmed that electropolished and non‐electropolished instruments within each group were equivalent in terms of geometry, cross‐sectional design, tip configuration, elemental composition, and phase transformation temperatures. Electropolishing significantly enhanced flexibility in both instrument types, as indicated by reduced bending loads and lower buckling resistance (*p* < 0.05). However, torsional strength was significantly reduced in the electropolished One RECI instruments, reflected by lower maximum torque and angle of rotation prior to fracture (*p* < 0.05). No significant torsional differences were observed in the One Curve mini group (*p* > 0.05). Surface microhardness and cutting efficiency remained unaffected by electropolishing in both systems (*p* > 0.05).

**Conclusions:**

Electropolishing improved the flexibility of both One RECI and One Curve mini NiTi instruments without compromising their surface microhardness or cutting efficiency. However, its impact on torsional resistance was system‐dependent, resulting in reduced strength only in the reciprocating One RECI instruments.

## 1 Introduction

The introduction of nickel‐titanium (NiTi) rotary instruments has revolutionised endodontic practice by improving the efficiency, safety, and predictability of root canal preparation (Bürklein and Arias 2022; Zupanc et al. 2018). Advances in metallurgy, design, and kinematics have produced instruments with enhanced flexibility, fatigue resistance, and cutting performance (Martins et al. 2022). Despite these innovations, NiTi instruments remain susceptible to unexpected fractures, particularly under challenging clinical conditions. Instrument separation not only complicates the procedure but may also compromise root canal disinfection and treatment outcomes (McGuigan et al. 2013; Ng et al. 2011). To overcome the limitations of conventional alloy processing, surface modification techniques such as electropolishing have been introduced.

Electropolishing is an electrochemical process that selectively removes surface material, producing a smooth, polished, and corrosion‐resistant finish (Chan et al. 2023; Praisarnti et al. 2010). When applied to NiTi alloys, commonly used in medical and dental devices, it offers several key advantages, including the removal of surface irregularities, burrs, and microdefects. This reduction in surface imperfections decreases friction, wear, and stress concentrators, thereby improving fatigue resistance (Mani et al. 2022). The process also forms a passive oxide layer, primarily TiO_2_ and NiO, which enhances corrosion resistance, an essential feature for devices exposed to physiological conditions (Wang et al. 2022). These properties are particularly relevant for NiTi endodontic instruments, which must withstand cyclic loading while maintaining mechanical integrity and biocompatibility. However, current evidence on the benefits of electropolishing in endodontics remains inconsistent. While some studies report enhanced mechanical performance (Anderson et al. 2007; da Silva et al. 2013; Lopes et al. 2010), others show limited or no improvements (Cheung et al. 2007; Peters et al. 2007). Moreover, most investigations focus primarily on cyclic fatigue resistance, with limited data on other important properties such as torsional strength, bending behaviour, buckling resistance, cutting efficiency, and hardness.

Two instruments that incorporate electropolishing as part of their manufacturing process are One RECI and One Curve mini (Micro‐Mega, Besançon, France). Both are made from heat‐treated C‐Wire NiTi alloy, which is purported to enhance flexibility and fatigue resistance (Goo et al. 2017). One RECI is a reciprocating instrument with an asymmetric cross‐section, designed to improve shaping efficiency and safety. One Curve mini, by contrast, is a rotary single‐file system developed to shape canals to full working length with minimal dentine removal. Although previous studies have compared their performance to other commercially available systems (Seracchiani et al. 2022; Silva et al. 2024), the specific contribution of electropolishing to their mechanical behaviour has not been systematically evaluated.

Determining whether electropolishing independently enhances the mechanical and structural properties of NiTi instruments requires experimental isolation of this variable. Only by comparing instruments with identical design, kinematics, and heat treatment, but differing in surface finishing, can the true effect of electropolishing be determined. Therefore, this study aimed to evaluate the impact of electropolishing on the mechanical performance of One RECI and One Curve mini‐instruments. Two versions of each instrument, identical in geometry and thermal treatment but differing by the presence or absence of electropolishing, were tested. The null hypothesis was that electropolishing would not significantly influence the mechanical properties of the evaluated instruments.

## 2 Material and Methods

The manuscript of this laboratory study has been written according to the Preferred Reporting Items for Laboratory Studies in Endodontology (PRILE) 2021 guidelines (Figure S1).

### 2.1 Sample Selection

To evaluate the isolated effect of surface electropolishing on the mechanical performance of endodontic NiTi instruments, two distinct types of instruments were selected based on their kinematics: a rotary instrument (One Curve mini 25/.06; Micro‐Mega, Besançon, France) and a reciprocating instrument (One RECI 25/.06; Micro‐Mega). A total of 212 instruments were specifically requested from the manufacturer for this study. For each instrument type, two sets were produced under identical conditions, differing only in the application of surface electropolishing, resulting in one electropolished and one non‐electropolished group per instrument type. Each subgroup consisted of 53 instruments, ensuring sufficient sample size for subsequent mechanical testing.

### 2.2 Design Analysis

Six instruments per group were randomly selected for microscopic design analysis. Images were captured under a dental operating microscope (Opmi Pico, Carl Zeiss Surgical, Jena, Germany) equipped with a camera (Canon EOS 500D; Canon, Tokyo, Japan), at 13.6× magnification with a light source. Specimens from both electropolished and non‐electropolished groups were imaged and compared in terms of active area length, number of spirals, and helical angles of the six most coronal spirals. Samples were then examined using a scanning electron microscope (SEM) (Hitachi S‐2400; Hitachi, Tokyo, Japan) at 20× magnification to confirm similarities in blade symmetry and tip geometry across groups. The instruments were examined in their original state to preserve surface characteristics and avoid any alteration that could affect the evaluation. Differences in surface finishing between electropolished and non‐electropolished instruments were also recorded at 150× and 200× magnification.

### 2.3 Metallurgical Assessment

The metallurgical properties were evaluated using energy‐dispersive X‐ray spectroscopy (EDS) and differential scanning calorimetry (DSC). For EDS analysis, three instruments per group were mounted on a sample holder and placed in the vacuum chamber of a SEM (Zeiss DSM 962; Carl Zeiss Microscopy GmbH, Munich, Germany) equipped with an Inca X‐act EDS detector (Oxford Instruments NanoAnalysis; Abingdon, UK). After a 5‐min vacuum stabilisation, measurements were taken at a 25 mm working distance, 3.1 A filament current, and 20 kV acceleration voltage. Data were acquired over 60 s with ~30% dead time, on a 400 μm × 400 μm active area. Number 5 processing time and ZAF correction were applied. Semi‐quantitative elemental composition was analysed using the Microanalysis Suite V4.14 software (Oxford Instruments NanoAnalysis; Abingdon, UK).

DSC testing followed the American Society for Testing and Materials guidelines (ASTM International 2004). A 4–5 mm fragment (5–10 mg) from the coronal active portion of each instrument was chemically etched for 2 min in a solution of 25% hydrofluoric acid, 45% nitric acid, and 30% distilled water, then neutralised in distilled water. Fragments were placed in an aluminium pan, with an empty pan used as reference, and tested in a DSC (DSC 204 F1 Phoenix; Netzsch‐Gerätebau GmbH, Selb, Germany). The thermal cycle lasted 1 h and 40 min, with a heating and cooling rate of 10°C/min across a temperature range from −150°C to 150°C. Tests were performed under a nitrogen atmosphere (20 mL/min). Heating was induced by internal resistors, while cooling was achieved using liquid nitrogen (120 mL/min). Phase transformation temperatures were determined using Netzsch Proteus Thermal Analysis software (Netzsch‐Gerätebau GmbH, Selb, Germany). A second instrument was tested for confirmation.

### 2.4 Mechanical Performance

Sample size calculation for each mechanical test was based on data obtained from the first five specimens, which were also included in the final analysis. Calculations were performed using a statistical power of 80%, a significance level (*α*) of 0.05, and the corresponding effect sizes and standard deviations derived from these initial trials. The required sample sizes for each test varied by instrument. For maximum torque, 23 specimens were needed for OneCurve mini (effect size ± SD = 0.06 ± 0.07) and 8 for OneReci (0.14 ± 0.09). For angle of rotation, due to high variability, OneCurve mini required 890 specimens (7.00 ± 52.7), while OneReci required 20 (41.4 ± 44.4). For maximum bending load, sample sizes were 20 for OneCurve mini (40.4 ± 24.4) and 7 for OneReci (29.6 ± 16.9). Buckling resistance showed lower variability, with 10 specimens needed for OneCurve mini (0.54 ± 0.40) and 7 for OneReci (0.58 ± 0.34). Cutting ability exhibited the greatest variability, especially in OneReci (0.8 ± 9.45), requiring 2191 specimens, compared to 153 for OneCurve mini (2.00 ± 6.24). For microhardness, the required sample sizes were 328 indentations for OneCurve mini (effect size ± SD = 18.0 ± 82.2) and 782 indentations for OneReci (7.4 ± 52.2), based on the initial five indentations.

Due to the considerable variability in the sample size calculations and the excessively large estimates in some tests—mainly driven by small effect sizes combined with the minimal standard deviations typical of highly standardised industrial samples—the calculated sample sizes often exceeded what would be practical or clinically relevant. To address this, a uniform and pragmatic sample size of 10 instruments per group was adopted for the mechanical tests, consistent with prior studies evaluating industrially manufactured NiTi instruments. For microhardness testing, a smaller sample size was used, limited to three instruments per group, with a total of 15 indentations performed per group to ensure reliable measurements while maintaining feasibility.

Torsional strength was tested using a static setup based on ISO 3630 and 3631 standards (ISO 3630‐3631 2008). Instruments were fixed straight on a TT100 torsiometer (Odeme Dental Research, Luzerna, Brazil), with the apical 3 mm clamped. They were rotated at 2 rpm (clockwise for One Curve mini and counterclockwise for One RECI) until fracture. Maximum torque (N·cm) and angle of rotation (°) at failure were recorded. Bending strength was also assessed according to ISO guidelines (ISO 3630‐3631 2008). Each instrument was held at a 45° downward angle, with the shaft in the motor's holder and the tip connected to a universal testing machine (Instron EMIC DL‐200 MF, São José dos Pinhais, Brazil). A 20 N load cell was used, attached to the machine that moved at 15 mm/min until the instrument bent to 45°. The force required was recorded in gram‐force (gf). Buckling strength was measured using a universal testing machine (Instron 4502; serial H3307, Bucks, England). Instruments were positioned vertically, tip down, on a marked metal base. The shaft was secured to the machine's head, connected to a 1‐kN load cell. A compressive force was applied at 1 mm/min until 1 mm of lateral deflection was reached (Lopes et al. 2012). The maximum buckling load was recorded in Newtons (N).

Microhardness was measured using a Vickers tester (Duramin; Struers Inc., Cleveland, OH, USA). Instruments were prepared according to ASTM guidelines (ASTM International 2023). Each instrument was embedded in acrylic resin and transversely sectioned at the middle third using a high‐speed diamond disc under constant irrigation. Microhardness was measured on the exposed cross‐sectional surface using a Vickers indenter, with a 100 gf load applied for 15 s (De‐Deus et al. 2017). Five indentations were made per instrument, and measurements were taken at 40× magnification. Results were recorded as Vickers Hardness Numbers (VHN). Cutting ability was tested using a custom setup combining an endodontic motor handpiece (AI‐Motor; Woodpecker, Guilin, China) with a universal testing machine fitted with a 500 N load cell. Each instrument was inserted into the coronal portion of a straight artificial root canal (size 15/0.02) embedded in a synthetic bone block (PCF 10; Sawbones, Vashon, WA, USA). A 10 gf preload was applied by the testing machine. One Curve mini was operated at 350 rpm with 2.5 N·cm torque in continuous clockwise rotation, while One RECI used a reciprocating motion (170° CCW/60° CW). The instrument advanced 3 mm and retracted 2 mm per cycle, progressing 1 mm with each stroke, until reaching a depth of 10 mm. The maximum force (gf) recorded during cutting was used to assess performance, with lower values indicating higher cutting efficiency.

### 2.5 Statistical Analysis

Mechanical parameter results are presented as means (with standard deviations) or medians (with interquartile range) to provide a comprehensive summary of the data distribution. All statistical analyses were conducted using SPSS version 28.0.0.0 (IBM SPSS Statistics, Chicago, IL, USA). Group comparisons were performed using either the independent samples *t*‐test or the Mann–Whitney *U*‐test, depending on data normality as assessed by the Shapiro–Wilk test. Statistical significance was set at *p* < 0.05.

## 3 Results

**FIGURE 1:**
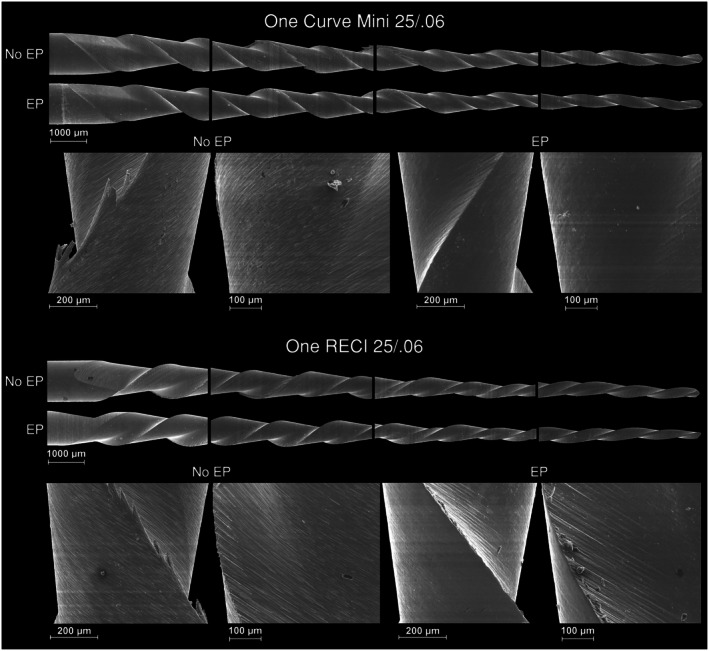
Representative scanning electron microscopy images of the tested NiTi instruments. Images on the top row illustrate the blade spiral design and tip geometry, revealing similar structural features between electropolished and non‐electropolished instruments within each system. The bottom row highlights surface topography. Although the visual differences may appear subtle, the electropolished instruments consistently exhibit smoother surfaces with fewer and less pronounced parallel machining marks when compared to their non‐electropolished counterparts. These features are indicative of a more uniform surface finish resulting from the electropolishing process.

**FIGURE 2:**
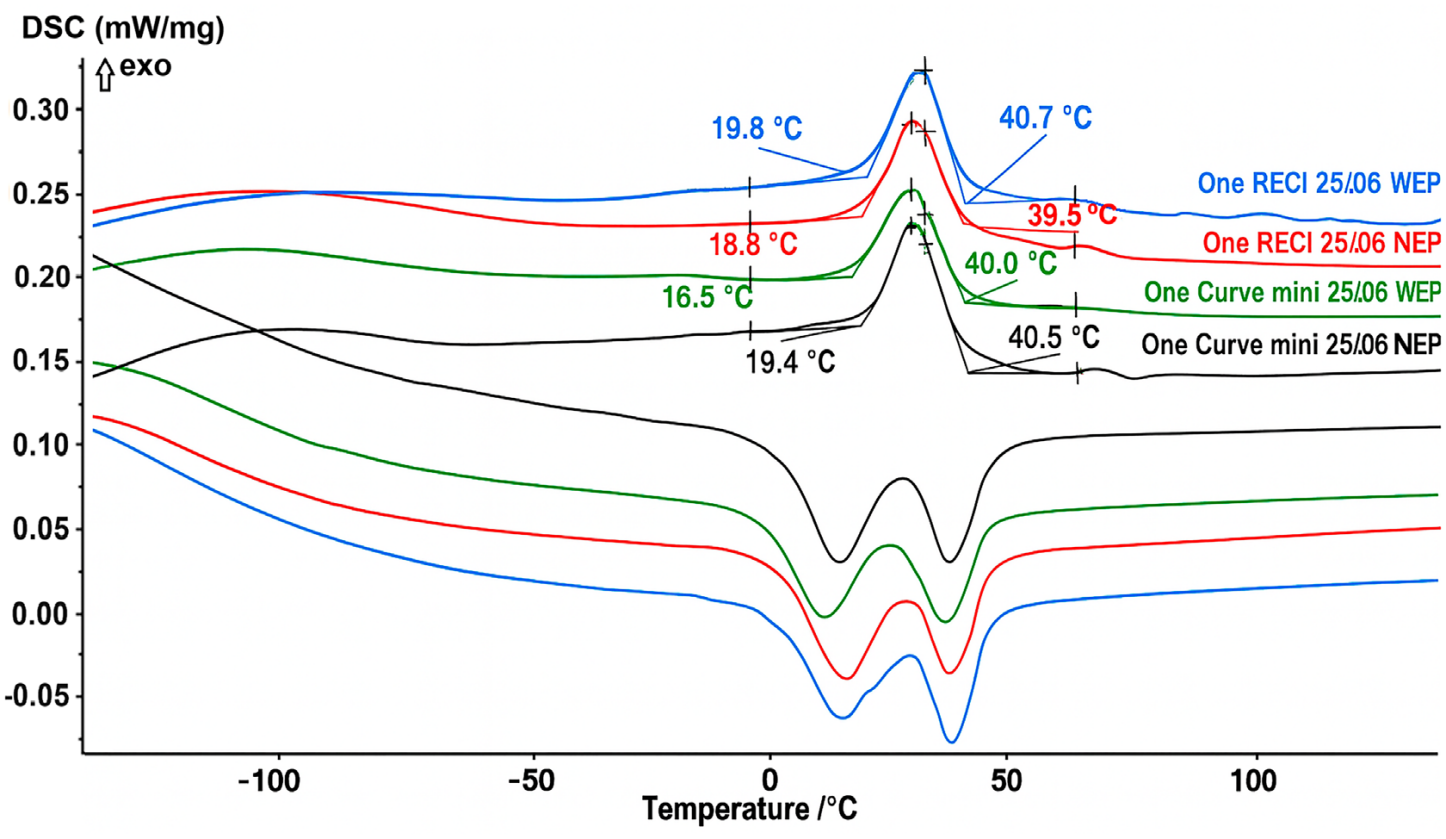
Differential scanning calorimetry curves demonstrating the phase transformation behaviour of the tested instruments. The heating (bottom) and cooling (top) curves confirmed that both electropolished and non‐electropolished versions of each instrument type exhibited comparable transformation temperatures. All groups displayed R‐phase start and finish temperatures around 40°C and 18°C, respectively. Similarly, all groups showed also an equivalent austenitic start and finish near to 0°C and 45°C, respectively. Cooling curves should be interpreted from right to left, and heating curves from left to right. These results confirm metallurgical consistency between groups, ensuring that electropolishing was the only variable under investigation.

The active area length, number of spirals, and helical angles were similar across groups for both One Curve mini (16 mm, 11 spirals, 33.4° ± 3.1° without vs. 33.1° ± 2.5° with electropolishing) and One RECI instruments (16 mm, 11 spirals, 34.7° ± 1.9° without vs. 35.1° ± 2.8° with electropolishing). SEM analysis confirmed comparable blade designs and tip geometries within each instrument type. Surface inspection revealed more blade irregularities and visible machining marks in non‐electropolished instruments, while electropolished instruments had smoother surfaces with fewer irregularities (Figure 1). Metallurgical analysis also confirmed group similarity. EDS showed a near‐equiatomic NiTi composition in all groups (One Curve mini: 1.024 without vs. 1.017 with electropolishing; One RECI: 1.054 without vs. 1.034 with electropolishing), with no other metallic elements detected. DSC results demonstrated similar phase transformation temperatures in all groups, with R‐phase start (Rs) near 40°C and finish (Rf) near 18°C, and austenite start (As) near 0°C and finish (Af) near 45°C (Figure 2).

For One Curve mini, electropolishing had no significant effect on maximum torsional strength (*p* = 0.631), angle of rotation (*p* = 0.653), microhardness (*p* = 0.412), or cutting ability (*p* = 0.783). However, electropolished instruments showed significantly lower bending strength (*p* = 0.001), indicating greater flexibility, and reduced buckling strength (*p* = 0.019) compared to non‐electropolished ones (Table 1). For One RECI, electropolishing significantly reduced maximum torque and angle of rotation (both *p* = 0.003), as well as bending and buckling strengths (both *p* = 0.001). In contrast, microhardness (*p* = 0.869) and cutting ability (*p* = 0.725) remained without statistical significance (Table 1).

**TABLE 1:** Mechanical behaviour results expressed as mean (standard deviation) and median (interquartile range).

Instrument	Torsional strength		Bending strength	Buckling	Microhardness (HVN)	Cutting ability (gf)
	Maximum torque (N·cm)	Angle of rotation (°)	Maximum load (gf)	Maximum load (*N*)		
One Curve mini 25/.06						
No electropolishing	0.66 ± 0.05 0.70 (0.60–0.70)	517.2 ± 36.8 517.0 (500.8–548.0)	354.2 ± 24.6 357.0 (335.7–374.3)	1.94 ± 0.36 1.90 (1.70–2.23)	416.9 ± 57.5 403.0 (390.0–429.0)	80.8 ± 6.7 81.5 (75.5–86.3)
With electropolishing	0.65 ± 0.07 0.60 (0.60–0.70)	507.2 ± 58.6 479.5 (472.3–562.0)	304.6 ± 15.1 309.5 (293.3–314.5)	1.59 ± 0.23 1.50 (1.48–1.83)	442.7 ± 69.5 420.0 (386.0–479.0)	81.6 ± 6.1 81.0 (78.0–85.5)
*p*	0.631^a^	0.653^b^	0.001^b^	0.019^b^	0.412^a^	0.783^b^
One RECI 25/.06						
No electropolishing	0.66 ± 0.07 0.65 (0.60–0.70)	445.2 ± 47.9 422.0 (412.5–466.0)	388.6 ± 20.9 386.0 (370.0–407.0)	2.06 ± 0.20 2.15 (1.87–2.20)	388.6 ± 36.6 377.0 (359.0–416.0)	111.9 ± 5.4 112.5 (108.3–115.8)
With electropolishing	0.55 ± 0.05 0.55 (0.50–0.60)	408.3 ± 35.1 401.0 (396.5–408.0)	348.9 ± 12.6 351.0 (345.3–358.8)	1.61 ± 0.15 1.65 (1.47–1.73)	392.3 ± 78.7 381.0 (330.0–482.0)	113.5 ± 13.1 112.5 (101.0–126.5)
*p*	0.003^a^	0.003^a^	0.001^b^	0.001^b^	0.869^b^	0.725^b^

^a^ Mann–Whitney *U*‐test.

^b^ Independent sample *t*‐test.

## 4 Discussion

This study aimed to investigate the impact of electropolishing on the mechanical properties of One RECI and One Curve mini NiTi instruments by directly comparing electropolished and non‐electropolished versions of each instrument, all produced with identical geometry and subjected to the same heat treatment. To ensure that electropolishing was the only variable affecting the outcomes, a comprehensive characterisation of the instruments was conducted. This included detailed assessments of design and metallurgical features to confirm true equivalence between groups, an essential step that is often overlooked in similar studies, where such equivalence is typically assumed based on manufacturer specifications alone. In the present investigation, optical microscopy, SEM, EDS, and DSC were employed to verify that all instruments within each group shared the same geometry, cross‐sectional shape, tip configuration, elemental composition, and phase transformation temperatures (Figures 1 and 2). Results from these tests confirmed that electropolishing was the only differing factor between groups, thereby strengthening the study's internal validity and allowing any observed differences in mechanical behaviour to be attributed solely to the surface treatment. Overall, the present findings indicate that electropolishing does not uniformly enhance mechanical performance across different systems, and the null hypothesis was therefore rejected.

The results demonstrated that electropolishing had a significant impact on the mechanical behaviour of both NiTi instruments tested, although the extent and nature of its effects varied according to the specific mechanical property and the type of instrument (Table 1). For both the One Curve mini (rotary) and One RECI (reciprocating) instruments, electropolishing consistently enhanced flexibility, as indicated by the lower loads recorded during bending tests. This increased flexibility was further corroborated by the buckling resistance results, with electropolished instruments showing reduced resistance to axial compression, a characteristic typically associated with more flexible instruments (Vieira et al. 2025). These outcomes suggest that electropolishing beneficially alters the surface topography, likely by reducing surface irregularities and residual stress concentrations, thereby facilitating improved elastic deformation and overall mechanical performance (Lopes et al. 2016; Praisarnti et al. 2010).

An interesting finding of this study was that the effect of electropolishing on torsional behaviour was not consistent across the two tested instruments (Table 1). The One RECI electropolished instruments exhibited a significant reduction in both maximum torque and angle of rotation prior to fracture, indicating decreased torsional strength. This reduction may be explained by multiple factors. Although electropolishing enhances surface smoothness and reduces microdefects (da Silva et al. 2013; Lopes et al. 2016), it can also result in the slight removal of superficial material (Anderson et al. 2007; Bui et al. 2008), potentially decreasing the effective cross‐sectional area and reducing the instrument's resistance to torsional stress. It may also be hypothesised that the increased flexibility conferred by electropolishing (Table 1) could also have altered the internal stress distribution under torsional loading, promoting earlier onset of plastic deformation and fracture. In contrast, the One Curve mini instruments did not exhibit any significant changes in torsional properties after electropolishing, a finding that aligns with previous observations by Barbosa et al. (2008), who reported that electrochemical polishing did not significantly affect the torsional resistance of K3 rotary instruments. Their study reinforces the notion that the influence of surface finishing on torsional performance may be limited or highly dependent on specific instrument design and alloy characteristics, suggesting that the effect of surface treatment on torsional resistance is system‐specific.

The variation observed in the torsional test likely reflects complex interactions among electropolishing, instrument geometry, and alloy properties under torsional stress. Although the tested instruments share similar manufacturing processes, subtle yet significant differences—such as variations in the initial diameter of the NiTi wire, baseline surface roughness, defect density, and residual machining marks—may influence the extent and uniformity of material removal during electropolishing and, consequently, the instrument's response to such surface modifications (Anderson et al. 2007; Lopes et al. 2016). Differences in core mass, cross‐sectional area, and metal distribution also affect how mechanical stresses are absorbed and dissipated during torsional loading, potentially modulating the instrument's sensitivity to changes in surface integrity (Zanza et al. 2021; Zhang et al. 2010). While the global phase composition and transformation temperatures were comparable among instruments, local differences in phase distribution, grain orientation, or work hardening, factors not easily detected by bulk techniques like DSC and EDS, may further contribute to the observed mechanical disparities (Zanza et al. 2021). These seemingly minor design and material variations could influence the impact of electropolishing on the mechanical performance of NiTi instruments, highlighting a need for further investigation in this largely unexplored area.

No significant differences in microhardness or cutting efficiency were observed between the electropolished and non‐electropolished versions of either instrument (Table 1). These findings indicate that electropolishing, while effective in smoothing surface irregularities, does not significantly affect cutting efficiency or the internal hardness of the NiTi alloy. In this study, microhardness was measured on cross‐sections, reflecting subsurface rather than surface properties. As confirmed by DSC and EDS analyses (Figure 2), the alloy's phase composition and crystalline structure remained unchanged between groups. Since electropolishing removes only a thin surface layer, it is unlikely to influence the material's bulk hardness. Likewise, cutting efficiency is influenced by a combination of factors, including tip sharpness, cross‐sectional design, rake angle, and flute configuration (McSpadden 2007), all of which were maintained across both groups. The absence of changes in these performance metrics supports the conclusion that electropolishing enhances surface quality without compromising either surface‐dependent characteristics, such as cutting capability or core mechanical integrity of the tested instruments (Bui et al. 2008). It functions as an electrochemical pickling process that does not alter the material's geometry, thereby preserving its original shape. Additionally, hardness is a key factor influencing the cutting ability of these instruments, and the preservation of this property further explains why the cutting performance remains unaffected.

Although this study offers a comprehensive evaluation of the mechanical performance of electropolished and non‐electropolished NiTi instruments, some limitations should be acknowledged. A primary limitation is the absence of cyclic fatigue testing, which was deliberately excluded due to the abundance of existing literature that has already addressed this aspect in detail (Anderson et al. 2007; Bui et al. 2008; da Silva et al. 2013; Lopes et al. 2010; Oh et al. 2010), providing a solid foundation of comparative data between electropolished and non‐electropolished instruments. Another limitation lies in the scope of the instrument selection, as only two specific types (One RECI and One Curve mini) were examined. While this focused approach allows for precise control and meaningful comparisons, it may limit the generalisability of the findings across the full spectrum of NiTi instruments currently available. Furthermore, the study did not assess the shaping ability of the instruments, a clinically relevant parameter that reflects how effectively an instrument can negotiate and conform to the complex anatomy of root canals. The absence of this evaluation leaves a gap in understanding how surface treatment might influence clinical performance. It is also important to acknowledge that non‐significant results in some parameters, such as microhardness and cutting efficiency, may be affected by limited statistical power. Although the sample size was justified by the low variability of industrial samples and aligned with precedents in similar studies, we recognise that certain effects might have remained undetected (type II error). These findings should thus be interpreted cautiously and verified in future research. Despite these limitations, the study presents several important strengths. A key strength is its rigorous experimental design, which ensured that the only variable between instrument groups was the presence or absence of electropolishing. By controlling all other manufacturing parameters such as geometry, heat treatment, and alloy composition, the study isolates the effect of electropolishing, minimising confounding factors and enhancing internal validity. Additionally, the use of a multimethod analytical approach, including mechanical testing, microscopy, and metallurgical characterisation, adds significant depth to the evaluation. This integrated methodology allows for a more reliable understanding of how electropolishing influences instrument behaviour under various mechanical stresses, thereby reinforcing the relevance of the findings.

## 5 Conclusions

Electropolishing had a significant yet system‐dependent impact on the mechanical behaviour of NiTi instruments. It consistently improved flexibility, evidenced by reduced bending load and buckling resistance, without adversely affecting internal microhardness or surface‐dependent cutting efficiency, indicating that the surface treatment does not compromise the intrinsic material properties or functional performance of the instruments. However, its impact on torsional resistance was system‐dependent, resulting in reduced strength only in the reciprocating One RECI instruments.

## Author Contributions

**Victor T. L. Vieira:** conceptualisation, analysis, experimental procedures, writing, review (lead); **Emmanuel J. N. L. Silva:** conceptualisation, analysis, experimental procedures, writing, review, and editing (lead); **Jorge N. R. Martins:** conceptualisation, analysis, experimental procedures, writing, review, and editing (lead); **Alejandro Jaime:** conceptualisation, analysis; **Carlos Garcia Puentes:** conceptualisation, analysis; **António Roma Torres:** conceptualisation, analysis; **Francisco Manuel Braz Fernandes:** experimental procedures; **Marco A. Versiani:** conceptualisation, analysis, experimental procedures, writing, review, and editing (lead); **Gustavo De‐Deus:** conceptualisation, analysis, experimental procedures, writing, review, and editing (lead); **Felipe G. Belladonna:** conceptualisation, analysis, experimental procedures, writing, review, and editing (lead).

## Ethics Statement

The authors have nothing to report.

## Conflicts of Interest

The authors declare no conflicts of interest.

## Supporting information


**Figure S1:** PRILE flowchart.

## Data Availability

Data available on request from the authors.
